# The Physician’s Mutual Benefit Assn. of Texas

**Published:** 1885-08

**Authors:** F. E. Daniel

**Affiliations:** Sec. and Treasurer


THE PHYSICIANS' MUTUAL BENEFIT ASSOCIATION OF TEXAS. —As inquiries are occasionally made about this organization it is stated for the benefit of all that the Comptroller has decided that this association, being purely benevolent, and having no capital invested, is not subject to the State tax. The membership is now sixty-five, consisting of some of the best known physicians of the States. The only expense attending membership is one dollar a year, payable on joining, and thereafter the first of January each year. In case a member dies, each surviving member will be called on to contribute one dollar to a fund for the deceased member's family. There are no other assessments for any purpose, and no other expense. There are no duties to be performed, except the payment of that one assessment of one dollar for each death. Applications for membership sent when requested, and certificates of membership issued on receipt of application and fee of one dollar.

F. E. DANIEL, M. D., Sec. and Treasurer.

